# Implication of serum copper level, serum zinc level, and copper to zinc ratio in neonatal sepsis

**DOI:** 10.1017/cts.2024.547

**Published:** 2024-10-24

**Authors:** Seyed Hossein Saadat, Rakhshaneh Goodarzi, Sadegh Kargarian Marvasti, Sobhan Montazerghaem

**Affiliations:** 1 Clinical Research Development Center of Children’s Hospital, Hormozgan University of Medical Sciences, Bandar Abbas, Iran; 2 Control and Prevention, Health Center of Fereydunshahr, University of Medical Sciences, Isfahan, Iran; 3 Student Research Committee, Faculty of Medicine, Hormozgan University of Medical Sciences, Bandar Abbas, Iran

**Keywords:** Zinc, copper, neonatal sepsis, trace elements, immune system

## Abstract

**Background:**

Zinc and copper are trace elements that have important roles in the function of the immune system. We aimed to compare serum zinc and copper levels in neonates with and without neonatal sepsis.

**Methods:**

This case–control study examined 54 newborns with sepsis and 54 matched healthy controls admitted to the neonatal intensive care unit of Children’s Hospital, Bandar Abbas, Iran. Neonates with the diagnosis of sepsis were regarded as cases and those admitted for other reasons were regarded as controls. Maternal and neonatal serum zinc and copper were measured on admission. Copper, zinc, and copper/zinc ratio differences between case and control groups were analyzed.

**Results:**

Neonatal zinc levels were significantly lower in the sepsis group versus controls (88.65 ± 40.64 vs 143.48 ± 69.57μg/dL, *p* < 0.001). Sepsis group mothers had lower zinc (66.04 vs 83.37μg/dL, *p* = 0.008) and copper (124.09 vs 157.74μg/dL, *p* < 0.001). Neonatal copper levels were slightly lower in the sepsis group. Copper/zinc ratio was significantly higher in the sepsis group (*p* < 0.001). In the sepsis group, the interval to the resolution of sepsis symptoms was significantly shorter in neonates with excess compared to sufficient copper levels (*P* = 0.023).

**Conclusions:**

Serum copper and zinc levels have an important role in the immune system’s response to the infection. Neonatal serum copper at levels higher than normal can lead to significantly shorter hospital stay. Also, higher Cu/Zn ratios can be found in neonatal sepsis, suggesting the potential utility of this index in the diagnosis of sepsis.

## Introduction

Neonatal sepsis is referred to the hemodynamic and clinical changes caused by a systemic viral, bacterial, or fungal infection and is associated with substantial morbidity and mortality [1]. With a global burden of 2202 per 100,000 live births, neonatal sepsis leads to an annual mortality rate of approximately 3 million neonates [2]. In a recent meta-analysis and systematic review, the prevalence of neonatal sepsis in Iran was estimated at 15.98% [3].

Zinc is a trace element that has a key role in the function of many biological enzymes [4,5]. Zinc also contributes to the normal function of the immune system, particularly cellular immunity, including the normal function of macrophages, neutrophils, and natural killer cells [6,7]. Studies suggest that immune function is adversely affected by impaired chemotaxis resulting from zinc deficiency. Moreover, higher serum zinc values have been associated with lower mortality and 48-hour sequential organ failure assessment score in adult patients with sepsis, reflecting the potential role of zinc in the immune response to sepsis [8]. Furthermore, higher than 75 µg/dL serum zinc levels have been reported to be correlated with a better prognosis of neonatal sepsis [9].

On the other hand, copper is another essential trace element, which is necessary for antioxidative defense and cellular metabolism [10,11]. Also, copper has a critical role in the maturation of the immune system, especially in the function of monocytes and neutrophils, as well as antibody production. Therefore, copper deficiency can predispose neonates to infectious disease [11,12]. However, copper levels appear to increase during infections as an acute phase reactant, while the redistribution of zinc into the liver and other tissues leads to a decrease in serum zinc levels [13,14]. As a result, copper to zinc (Cu/Zn) ratio has been proposed as a promising diagnostic biomarker in early-onset neonatal infections [14].

In this study, we aimed to compare serum zinc and copper levels between neonates with and without neonatal sepsis.

## Methods

### Participants

In this case–control study, neonates admitted to Bandar Abbas Children’s Hospital were evaluated. Inclusion criteria were gestational age (GA) ≥ 37 weeks, age < 29 days, and admission to the neonatal intensive care unit (NICU), and exclusion criteria were apparent congenital anomalies, asphyxia and resuscitation at birth, and any signs or symptoms indicative of chromosomal abnormalities for both cases and controls. Cases were diagnosed with sepsis based on the following criteria [15]:

- Presence of at least two clinical signs including:
  - Pallor or generalized jaundice
  - Cardiovascular manifestations: tachycardia or bradycardia, poor perfusion, or shock
  - Change in body temperature (hypo- or hyperthermia)
  - Respiratory manifestations: grunting, intercostal retraction, apnea, or cyanosis
  - Neurological manifestations: hypotonia, lethargy, irritability, or seizure
  - Gastrointestinal manifestations: abdominal distension, hepatosplenomegaly
- C-reactive protein (CRP) > 5 mg/dl
- At least one positive laboratory parameter including:
  - White blood cell count < 5000/µl or > 25000/µl at birth, or > 30000/µl 12–24 postnatal, or > 21000 on the second day or thereafter
  - Absolute neutrophil count < 1800/µl
  - Immature/total neutrophil ratio (I/T) < 0.16 on the first day or < 0.13 on the second day or thereafter

Neonates admitted to the NICU for any reason other than sepsis (mostly neonatal jaundice) were included in the control group. In other words, neonates admitted to the NICU for any reason other than sepsis, including those with jaundice, were included in the control group. Neonates of the control group who developed any signs/symptoms and laboratory findings of sepsis during the study were excluded.

The sample size was calculated as 54 patients in each group (case or control), based on the difference of mean serum zinc levels between neonates with and without sepsis in the study by Visalakshy *et al*. [8], using *α* = 0.05 and *β* = 0.2.

### Study design

Age, gender, GA, admission weight, as well as birth weight, head circumference (HC), and height were recorded for neonates of both groups. Length of hospital stay, the frequency of antibiotic regimen change, and the interval to the resolution of sepsis symptoms were noted in the sepsis group (cases). A 2.5-ml random blood sample was collected on admission form the neonates and their mothers in both groups for the measurement of serum zinc and copper. Serum zinc and copper levels were measured again on the 5^th^ day of admission in the sepsis group. Serum copper was measured with colorimetric analysis using the Biorexfars kit (Biorexfars Co., Iran). Serum zinc was measured with end-point detection method using the Archem Diagnostics kit (Archem Diagnostics Ltd., Turkey). Cu/Zn ratio was calculated for both groups. Serum zinc levels of 80–120 µg/dL were regarded as normal for both mothers and children. Serum copper levels of 20–70 µg/dL were regarded as normal for neonates and 80–155 µg/dL for mothers [16].

### Data analysis

We used the Statistical Package for the Social Sciences (SPSS) software (version 26.0, Armonk, NY: IBM Corp.) for data analysis. Quantitative data were described using means and standard deviations and qualitative data using frequencies and percentages. Chi-square test was used to compare qualitative variables between cases and controls. Based on the results of the Kolmogorov–Smirnov normality test, either Mann–Whitney test or independent *t*-test were used for the comparison of quantitative variables between groups. Also, one-way analysis of variance (ANOVA) or Kruskal-Wallis tests were used to compare quantitative variables when there were more than two groups regarding maternal copper levels. To compare serum zinc and copper levels in the sepsis group on admission and on the 5^th^ day of hospitalization, paired *t*-test and Wilcoxon test were used based on the distribution normality. P-values ≤ 0.05 were regarded as statistically significant.

## Results

From the total 108 neonates included in this study with a mean age of 8.79 ± 7.28 days, 67 (62%) were male and 41 (38%) were female. Table 1 shows the comparison of general characteristics between cases and controls. Neonates of the sepsis and control group were comparable in terms of age, gender, GA, admission weight, birth weight, HC, and height.

**Table 1.** General characteristics of the study population in cases and controls

Variables	Total (*n* = 108)	Sepsis (*n* = 54)	Control (*n* = 54)	*P*-value
Age (days) mean ± SD	8.79 ± 7.28	10.89 ± 8.62	6.69 ± 4.88	0.157‡
Gender *N* (%)				
Male	67 (62.0)	37 (68.5)	30 (55.6)	0.165*
Female	41 (38.0)	17 (31.5)	24 (44.4)	
GA (weeks) mean ± SD	38.40 ± 1.00	38.37 ± 1.07	38.43 ± 0.94	0.802‡
Admission weight (g) mean ± SD	3056.02 ± 443.86	3111.67 ± 498.66	3000.37 ± 377.86	0.194†
Birth weight (g) mean ± SD	3083.66 ± 375.17	3066.39 ± 392.22	3100.93 ± 360.15	0.635†
HC (cm) mean ± SD	34.41 ± 1.22	34.20 ± 0.98	34.61 ± 1.39	0.224‡
Height (cm) mean ± SD	49.32 ± 1.63	49.15 ± 1.61	49.50 ± 1.66	0.251‡

N = number; SD = standard deviation; GA = gestational age; HC = head circumference.

^*^Analyzed by chi-square test.

^†^Analyzed by independent *t*-test.

^‡^Analyzed by Mann–Whitney test.

Maternal and neonatal serum zinc and copper levels on admission were lower in the sepsis group compared to controls; however, the difference between cases and controls regarding neonatal serum copper levels was not statistically significant (*P* = 0.109). Moreover, neonatal Cu/Zn ratio was significantly higher in the sepsis group compared to controls (*P* < 0.001). By subtracting the neonatal serum zinc and copper levels from their maternal levels we found a gap which shows significantly lower difference between maternal and neonatal zinc and copper levels in the sepsis group (*P* = 0.001 and *P* = 0.024, respectively) (Table 2). This approach allowed us to identify any significant gaps that could potentially contribute to the development of neonatal sepsis, particularly in neonates with normal serum levels but a significant gap with their mothers.

**Table 2.** Comparison of neonatal and maternal serum zinc and copper levels between cases and controls on the admission day

Variables	Total (*n* = 108)	Sepsis (*n* = 54)	Control (*n* = 54)	*P*-value*
Maternal Zn (µg/dL) mean ± SD	74.70 ± 34.64	66.04 ± 26.65	83.37 ± 39.48	0.008
Maternal Cu (µg/dL) mean ± SD	140.92 ± 50.82	124.09 ± 37.40	157.74 ± 56.90	<0.001†
Neonatal Zn (µg/dL) mean ± SD	143.48 ± 69.57	88.65 ± 40.64	143.48 ± 69.66	<0.001
Neonatal Cu (µg/dL) mean ± SD	83.19 ± 33.73	72.07 ± 26.11	83.19 ± 33.73	0.109
Neonatal Cu/Zn ratio mean ± SD	0.80 ± 0.45	0.93 ± 0.41	0.67 ± 0.45	<0.001
Maternal Zn – neonatal Zn (µg/dL) mean ± SD	−41.36 ± 63.39	−22.61 ± 41.54	−60.11 ± 75.30	0.001
Maternal Cu – neonatal Cu (µg/dL) mean ± SD	63.29 ± 51.93	52.02 ± 38.09	74.56 ± 61.12	0.024†

N = number; SD = standard deviation; Zn = zinc; Cu = copper; Cu/Zn = copper to zinc ratio.

^*^Analyzed by Mann–Whitney test.

^†^Analyzed by independent *t*-test.

In the sepsis group, 77.8% of the mothers had deficient serum zinc levels while in the control group the corresponding percentage was 63% (*P* = 0.092). Comparison of neonatal serum zinc levels based on the sufficiency of maternal zinc (Table 3) showed that neonatal serum zinc levels were significantly higher when maternal zinc was sufficient compared to when in was deficient in both cases and controls (*P* = 0.008 and *P* = 0.016, respectively). Also, there was a significant difference between the sepsis group and controls regarding neonatal zinc levels when mothers had zinc deficiency (80.40 ± 36.59 vs. 125.62 ± 56.71, *P* < 0.001); however, although neonatal zinc levels were higher in controls with sufficient maternal zinc compared to their counterparts in the sepsis group, the difference was not statistically significant (P = 0.064).

**Table 3.** Correlation of maternal zinc and copper with neonatal zinc and copper levels

Variables	Total (*n* = 108)	Sepsis (*n* = 54)	Control (*n* = 54)	*P*=value
Maternal Zn *N* (%)				
Deficient	76 (70.4)	42 (77.8)	34 (63.0)	0.092*
Sufficient	32 (29.6)	12 (22.2)	20 (37.0)	
Neonatal Zn (µg/dL) mean ± SD				
Deficient maternal Zn	100.63 ± 51.57	80.40 ± 36.59	125.62 ± 56.71	<0.001†
Sufficient maternal Zn	152.72 ± 73.03	117.50 ± 42.40	173.85 ± 80.05	0.064†
P-value	<0.001†	0.008†	0.016†	
Maternal Cu *N* (%)				
Deficient	10 (9.3)	6 (11.1)	4 (7.4)	0.028*
Sufficient	63 (58.3)	37 (68.5)	26 (48.1)	
Excess	35 (32.4)	11 (20.4)	24 (44.4)	
Neonatal Cu (µg/dL) mean ± SD				
Deficient maternal Cu	61.10 ± 26.43	53.83 ± 20.84	72.00 ± 33.30	0.314¶
Sufficient maternal Cu	75.87 ± 27.79	71.32 ± 22.52	82.35 ± 33.34	0.150¶
Excess maternal Cu	85.51 ± 34.50	84.55 ± 34.61	85.96 ± 35.19	0.903†
P-value	0.054‡	0.110§	0.564‡	

N = number; SD = standard deviation; Zn = zinc.

^*^Analyzed by chi-square test.

^†^Analyzed by Mann–Whitney test.

^‡^Analyzed by Kruskal–Wallis test.

^§^Analyzed by one-way ANOVA test.

^¶^Analyzed by independent *t*-test.

Although the percentage of mothers with deficient copper levels was significantly higher in the sepsis group compared to controls (11.1% vs. 7.4%, *P* = 0.028), there was no statistically significant difference regarding neonatal copper levels among neonates from mothers with deficient, sufficient, and excess copper levels in neither of the groups. Moreover, in the three groups of mothers with various copper sufficiency, no significant difference was found between cases and controls in terms of neonatal copper levels (Table 3). We additionally assessed zinc and copper levels on the 5th day of hospitalization for all neonates diagnosed with sepsis. This follow-up measurement was crucial to determine whether there were significant changes in these trace element levels over the course of treatment. This aspect of our study design aimed to provide deeper insights into the dynamic fluctuations of zinc and copper levels in response to sepsis and its treatment. Despite the slight increase in neonatal serum zinc and copper levels from admission to the 5^th^ day of hospital stay, Cu/Zn ratios remained the same in the sepsis group (Table 4).

**Table 4.** Serum zinc and copper levels in the sepsis group on admission and on the 5^th^ day

Variables	Admission	5^th^ day	P-value
Neonatal Zn (µg/dL) mean ± SD	88.65 ± 40.64	94.41 ± 38.151	0.582†
Neonatal Cu (µg/dL) mean ± SD	72.07 ± 26.11	79.35 ± 32.25	0.267†
Neonatal Cu/Zn ratio mean ± SD	0.93 ± 0.41	0.93 ± 0.41	0.984*

N = number; SD = standard deviation; Cu = copper; Zn = zinc; Cu/Zn = copper to zinc ratio.

^*^Analyzed by paired *t*-test.

^†^Analyzed by Wilcoxon test.

The mean length of hospital stay was 10.87 ± 5.76 days in the sepsis group and the mean interval to the resolution of sepsis symptoms was 3.24 ± 3.29 days. The antibiotic regimen did not change in 34 patients (63%) in the sepsis group, while it changed once in 8 (14.8%), twice in 9 (16.7%), and three times in (5.6%). The interval to the resolution of sepsis symptoms was significantly shorter in neonates with excess compared to sufficient copper levels (*P* = 0.023) (Table 5).

**Table 5.** In-hospital variables by neonatal zinc and copper sufficiency levels in the sepsis group

Variables	Deficient Zn (*n* = 27)	Sufficient Zn (*n* = 13)	Excess Zn (*n* = 14)	*P*-value*	Sufficient Cu (*n* = 32)	Excess Cu (*n* = 22)	*P*-value†
Hospital length of stay (days) mean ± SD	10.78 ± 5.69	12.31 ± 7.64	9.71 ± 3.63	0.742	11.78 ± 6.82	9.55 ± 3.46	0.217
Interval to symptom resolution (days) mean ± SD	3.07 ± 1.71	4.00 ± 5.07	2.86 ± 3.70	0.229	3.59 ± 3.28	2.73 ± 3.30	0.023
Frequency of antibiotic regimen change mean ± SD	0.78 ± 1.05	0.69 ± 0.95	0.36 ± 0.75	0.389	0.78 ± 1.01	0.45 ± 0.86	0.202

N = number; SD = standard deviation; Cu = copper; Zn = zinc.

^*^Analyzed by Kruskal–Wallis test.

^†^Analyzed by Mann–Whitney test.

## Discussion

We found neonatal serum zinc levels to be significantly lower in patients with neonatal sepsis compared to controls. We also found lower serum copper levels in neonatal sepsis; however, the difference between cases and controls was not statistically significant. Similarly, Wang et al. reported significantly lower serum zinc levels in ill neonates compared to controls and found no significant difference regarding serum copper levels [17]. Nevertheless, ill neonates were defined by the score for neonatal acute physiology (SNAP) in their study, which includes 34 parameters and is different from sepsis criteria. Wisniewska et al.’s findings were also consistent with our results as they showed significantly lower serum zinc levels in neonates with early-onset congenital infections compared to controls along with comparable serum copper levels [14]. Their results regarding Cu/Zn ratio was also in line with ours. In our study, Cu/Zn ratio was significantly higher in patients with neonatal sepsis compared to controls. Likewise, Wisniewska et al. found significantly higher Cu/Zn ratio in neonates with early-onset infections [14]. The higher Cu/Zn ratio in neonatal sepsis can be justified by the increase in serum copper in response to acute infection coupled with a relative decrease in serum zinc due to its redistribution into the liver and other tissues [18–20]. Furthermore, in agreement with our results, Wahby et al. demonstrated significantly lower zinc and copper levels in neonates diagnosed with sepsis in comparison with the control group. They proposed neonatal serum zinc and copper levels as relevant risk factors for neonatal sepsis as well, with serum copper being a stronger indicator of neonatal sepsis compared to zinc [21]. Their higher sample size could have increased the difference between the sepsis and control groups regarding serum copper to a significant level.

Another finding of our study was that maternal serum zinc and copper levels were significantly lower in the sepsis group compared to controls. Previous studies have demonstrated that maternal copper and zinc levels are directly correlated with their neonatal levels [22]. Also, copper and zinc deficiency in mothers has been associated with neonatal sepsis [23–25]. This has been confirmed in a meta-analysis of 17 randomized controlled trials including more than 9000 women and their children [26]. Nevertheless, a more recent meta-analysis, including 54 trials on over 17,000 women and their babies showed no correlation between maternal zinc levels and neonatal sepsis [27]. To show this we divided maternal zinc and copper levels into deficient, sufficient, and excess groups based on the normal reference range and compared neonatal zinc and copper levels accordingly. The results showed that zinc levels were significantly higher in neonates of mothers with sufficient zinc compared to those of mothers with deficient zinc in both the sepsis and control groups. The same trend was observed with copper levels; however, the correlation between maternal and neonatal copper levels was not statistically significant.

By comparing neonatal zinc and copper levels in the sepsis group on admission and on the 5^th^ day of hospitalization, we found an increase, although statistically insignificant, in the mean levels of both trace elements. The same results have been reported in our previous pilot study [28]. Another finding of our study was that neonates in the sepsis group with excess serum copper levels had significantly shorter length of hospital stay compared to those with copper levels within the normal range. Nevertheless, this can be due a stronger acute phase response in neonates with excess copper since copper levels had been measured after the diagnosis of sepsis [29].

The major strength of our study was that patients in the sepsis and control groups were comparable with respect to the potential effectors or confounding factors, including birth and admission weight, age, GA, height, and HC. Another strength of the current study was the simultaneous evaluation of maternal zinc and copper levels which had not been taken into account in a similar manner in previous studies.

Our study was not without limitations. One limitation of the current study was that we assessed serum zinc and copper levels when the neonates had already developed sepsis. The results in the sepsis group can merely reflect these levels in response to infection. A prospective study design evaluating the baseline zinc and copper levels before the development of sepsis would yield better results.

In addition, we recognize certain methodological limitations that could have implications for the interpretation of our findings. Notably, the absence of data on breastfeeding practices, anemia prevalence, and leukocytosis/leukopenia in our study participants limits our ability to fully contextualize the observed zinc and copper serum levels within the broader spectrum of neonatal health. These parameters, significant in their potential influence on neonatal outcomes, present a challenge in data collection, particularly in neonatal populations. Furthermore, our study’s exclusion of iron and selenium level assessments – elements integral to immune system functionality and potentially impactful in sepsis outcomes [30] – denotes a limitation in our current scope, underscoring the necessity for future research endeavors to incorporate a more comprehensive array of trace elements. Additionally, the relatively small sample size of our cohort may constrain the generalizability and robustness of our conclusions. It is imperative for subsequent studies to engage larger and more diverse neonatal populations, thereby enhancing the reliability and applicability of findings in the complex interplay of trace elements and neonatal sepsis.

## Conclusions

In our study, neonates with sepsis exhibited significantly lower serum zinc levels compared to controls. Additionally, while serum copper levels were also lower in the sepsis group, this difference did not reach statistical significance. Moreover, mothers of neonates in the sepsis group had significantly lower serum zinc and copper levels. This shows the importance of zinc and copper sufficiency in both mothers and their babies for the prevention of neonatal sepsis. However, these results, especially regarding neonatal and maternal copper levels in association with sepsis, should be confirmed in future studies with a larger sample size and potentially different study design.

## Data Availability

The datasets used and/or analyzed during the current study are available from the corresponding author on reasonable request.
